# Prompt-Induced Output Variability and Structured-Output Integrity in Local Open Large Language Models: A Multi-model In Silico Benchmark Using Synthetic Acute-Care Scenarios

**DOI:** 10.7759/cureus.108768

**Published:** 2026-05-13

**Authors:** Yuusuke Harada

**Affiliations:** 1 Graduate School of Public Policy, Hosei University, Tokyo, JPN; 2 Graduate School of Humanities and Social Sciences, Hiroshima University, Hiroshima, JPN

**Keywords:** clinical reasoning, jensen-shannon distance, large language models, local models, medical informatics, prompt sensitivity, robustness, synthetic cases

## Abstract

Background

Large language models (LLMs) are increasingly evaluated for clinical knowledge retrieval, documentation support, triage, diagnostic assistance, and conversational decision support. However, high benchmark performance does not ensure stable behavior under realistic workflow conditions. We evaluated whether ordinary prompt variation, without explicit adversarial forcing, produces measurable output divergence in local open LLMs when applied to synthetic acute-care scenarios. This study assessed prompt-induced output variability and structured-output integrity, not clinical correctness, management appropriateness, or patient safety.

Methods

We performed a descriptive in silico benchmark using three local open models served through Ollama (Ollama, Palo Alto, CA, USA): DeepSeek-R1 14B (DeepSeek, Hangzhou, China), Gemma 3 12B (Google DeepMind, London, UK), and gpt-oss 20B (OpenAI, San Francisco, CA, USA). Six synthetic acute-care cases were presented across three sequential timepoints. At each timepoint, models returned structured JSON containing probability distributions over a fixed 10-diagnosis ontology and an eight-action management ontology. We tested 21 prompt variants, three repeated benchmark runs, and three prompt-layer conditions: a baseline condition (BASE), a stability-oriented condition (STABLE), and WOBBLEv2. Primary analyses used pair-valid comparisons, in which each perturbed prompt was compared with its matched baseline prompt only when both outputs were valid. Divergence was quantified with the Jensen-Shannon distance for diagnosis and action distributions and summarized as the mean total Jensen-Shannon distance. Structured-output integrity was assessed using invalid output rate and agreement between the self-declared top action and the argmax of the returned action-probability vector.

Results

Across the nine model-condition cells, the mean total Jensen-Shannon distance ranged from 0.126 to 0.306. The lowest mean divergence was observed for Gemma 3 12B under STABLE, whereas the highest was observed for DeepSeek-R1 14B under BASE. Action distributions were more labile than diagnosis distributions in every model-condition cell. At the terminal timepoint, top-action switching ranged from 0.253 to 0.584. STABLE reduced the mean total Jensen-Shannon distance substantially for Gemma 3 12B but only minimally for DeepSeek-R1 14B and gpt-oss 20B. The mean total Jensen-Shannon distance was higher at the terminal timepoint than at the first timepoint in all nine cells. Integrity profiles differed by model: Gemma 3 12B produced no invalid outputs in the primary analysis set but had low top-action argmax agreement at later timepoints, whereas gpt-oss 20B maintained high argmax agreement among valid outputs but had the highest invalid output rates.

Conclusions

Within this fixed synthetic benchmark, stable prompt-response behavior did not generalize uniformly across local model families. Ordinary prompt variation was sufficient to produce measurable output divergence under both BASE and STABLE conditions, and management-action distributions were consistently more prompt-sensitive than diagnosis distributions. These findings support reporting distributional stability and structured-output integrity as separate endpoints in local LLM robustness audits. They should not be interpreted as evidence of clinical accuracy, clinical error, or patient-safety impact.

## Introduction

Large language models (LLMs) are increasingly studied for clinical knowledge retrieval, documentation support, triage, diagnostic assistance, and conversational decision support [1-5]. High benchmark accuracy, however, does not ensure robust behavior under realistic clinical workflow conditions. In medicine, small changes in wording, order of presentation, tone, urgency, or audience framing may shift a recommendation even when the underlying clinical facts are unchanged.

Prompt robustness has therefore become an important evaluation target in LLM research [6,7]. In medicine, robustness must be understood more broadly than resistance to adversarial attacks. Clinical deployment also depends on whether sensitivity appears under ordinary prompts, whether it affects management more than diagnosis, whether it accumulates across sequential interactions, and whether structured outputs remain intact when ambiguity, urgency, or dialogue context is present. These questions are especially relevant for local open-model deployments, which may support privacy-preserving inference, hospital-side customization, and lower marginal cost.

Recent clinical evaluations have moved beyond licensing-style multiple-choice tasks toward more realistic, open-ended settings. Studies of case-based decision-making, together with reproducibility, robustness, and uncertainty-focused evaluations, show that performance may change when models must gather information iteratively, follow guidelines, communicate uncertainty, or produce management plans rather than select a final diagnosis [8-21]. Trustworthy medical artificial intelligence evaluation should therefore include calibration, uncertainty communication, reproducibility, and workflow-facing failure modes, not accuracy alone. However, limited empirical work has examined how local open models behave when the same clinical information is delivered through minimally different ordinary prompts across multiple turns.

We therefore performed an in silico benchmark study of prompt sensitivity in a local multi-model setting. The primary problem addressed in this study was whether ordinary prompt variation, without explicit adversarial forcing, changes structured diagnostic and management outputs in local open models.

The objectives were to (1) quantify prompt-induced divergence across local open models under the baseline condition (BASE) and a stability-oriented condition (STABLE), (2) compare the sensitivity of diagnosis distributions with the sensitivity of management-action distributions, and (3) evaluate structured-output integrity as a second robustness endpoint.

We hypothesized that measurable prompt sensitivity would be detectable without explicit adversarial forcing, that action distributions would be more labile than diagnosis distributions, and that different model families would display distinct integrity failure modes. The goal was not to assess clinical correctness, management appropriateness, or patient safety, but to characterize the stability of output distributions under ordinary prompt variation within a fixed synthetic benchmark.

## Materials and methods

Study design and benchmark scope

**Figure 1:**
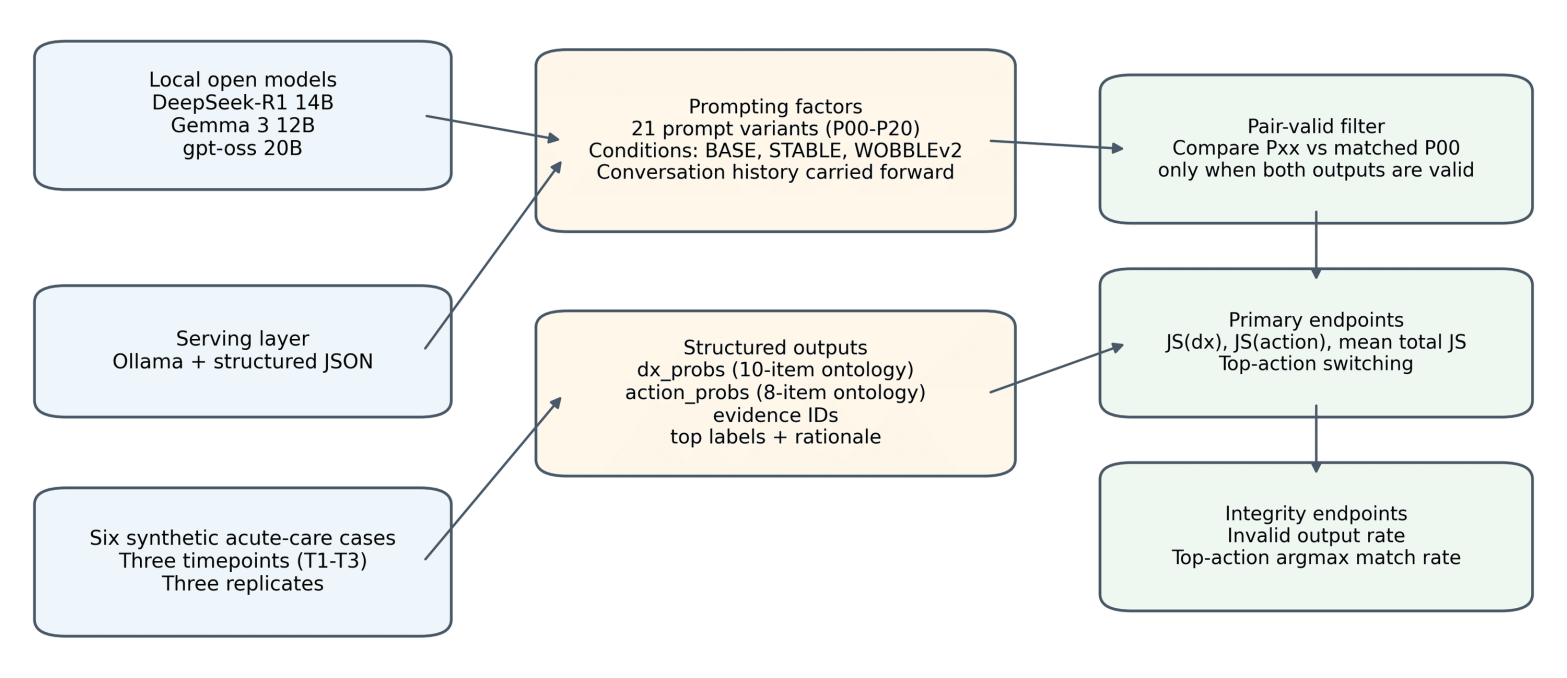
Benchmark design and analysis workflow. Schematic of the local multi-model benchmark. Three local models were served through Ollama and evaluated on six synthetic acute-care cases revealed across three sequential timepoints. For each case and timepoint, 21 prompt variants and three prompt-layer conditions were applied. Outputs were constrained to structured JSON containing diagnosis probabilities, action probabilities, evidence identifiers, and top-label fields. Pair-valid analyses compared each perturbed prompt only with its matched P00 baseline when both outputs were valid. Primary endpoints were diagnosis Jensen-Shannon distance, action Jensen-Shannon distance, mean total Jensen-Shannon distance, and top-action switching; integrity endpoints were invalid output rate and top-action argmax match rate.

This was an in silico local multi-model benchmark using synthetic acute-care cases only. Figure 1 summarizes the benchmark design and analysis workflow.

The primary analysis set comprised six cases: pneumonia progressing toward sepsis, pulmonary embolism progressing toward obstructive shock, acute heart failure with chronic obstructive pulmonary disease overlap, COVID-19 with worsening hypoxemia, pulmonary tuberculosis with worsening respiratory compromise, and acute coronary syndrome with pulmonary edema or shock physiology. Each case unfolded across three sequential timepoints. No human participants, identifiable patient information, or real clinical records were used. Institutional review board review was therefore not required.

Models and serving environment

The benchmark was executed through Ollama version 0.23.1 (Ollama, Palo Alto, CA, USA) on a local workstation. Three local open models were evaluated with schema-constrained structured outputs: DeepSeek-R1 14B (deepseek-r1:14b) (DeepSeek, Hangzhou, China), Gemma 3 12B (gemma3:12b) (Google DeepMind, London, UK), and gpt-oss 20B (gpt-oss:20b) (OpenAI, San Francisco, CA, USA). The experiment configuration used temperature 0.2, top-p 0.95, a maximum output length of 1,000 tokens, and three repeated benchmark runs identified as R1, R2, and R3. The raw Ollama model tags and installed-model digests were as follows: `deepseek-r1:14b`, digest `c333b7232bdb521236694ffbb5f5a6b11cc45d98e9142c73123b670fca400b09`; `gemma3:12b`, digest `f4031aab637d1ffa37b42570452ae0e4fad0314754d17ded67322e4b95836f8a`; and `gpt-oss:20b`, digest `17052f91a42e97930aa6e28a6c6c06a983e6a58dbb00434885a0cf5313e376f7`. All three evaluated models were in GGML Unified Format (GGUF). The installed parameter sizes and quantization levels were as follows: DeepSeek-R1 14B, 14.8B parameters, Q4_K_M; Gemma 3 12B, 12.2B parameters, Q4_K_M; and gpt-oss 20B, 20.9B parameters, microscaling 4-bit floating-point format (MXFP4). The benchmark was executed on a local workstation with an Intel® Core™ i9-14900 CPU (24 cores/32 threads), 64 GB RAM, and an NVIDIA RTX 2000 Ada Generation graphics processing unit (GPU) with 16 GB video random access memory (VRAM). An integrated Intel UHD Graphics 770 GPU was also present. Per-token GPU-offload traces were not separately logged. The orchestration layer attempted seed control with identifiers 101, 202, and 303 where supported by the local serving stack; however, analyses treated replicates as repeated benchmark runs rather than as inferential samples. Conversation history was carried across timepoints so that each model experienced the case as a sequential dialogue rather than as isolated snapshots. The observed results therefore reflect the joint behavior of the model, local serving stack, prompt template, and structured-output constraint.

Ontology and output schema

At each timepoint, every model was asked to return valid JSON containing diagnosis probabilities over a fixed 10-item ontology, action probabilities over a fixed eight-item ontology, evidence fact identifiers, and brief rationale text. The diagnosis ontology covered community-acquired pneumonia, pulmonary embolism, acute heart failure, chronic obstructive pulmonary disease exacerbation, COVID-19 infection, sepsis, pneumothorax, asthma exacerbation, tuberculosis, and acute coronary syndrome. The action ontology covered empiric antibiotics, CT pulmonary angiography, general-ward admission, supplemental oxygen, intravenous fluids, vasopressors, intensive care unit transfer, and close reassessment or monitoring.

Prompt variants and prompt-layer conditions

We evaluated 21 prompt variants. P00 was the neutral baseline prompt. The remaining variants introduced light perturbations across politeness, punctuation, persona, risk tolerance, tone, evidence policy, audience framing, urgency, verbosity, and focus. Three prompt-layer conditions were included in the primary manuscript dataset: BASE, STABLE, and WOBBLEv2. STABLE was designed to reduce stylistic variability and encourage evidence-grounded reporting. WOBBLEv2 served as an internal audit condition for discrete mode switching and was not interpreted as evidence of intrinsic model dynamics. The full system prompt, user prompt template, prompt-layer condition texts, all 21 prompt variants (P00-P20), the diagnosis/action ontology, the JSON output schema, and validation scripts are available in the Zenodo reproducibility archive at https://doi.org/10.5281/zenodo.20079035.

Analysis policy and metrics

Divergence was evaluated only for pair-valid comparisons. For a given model, case, condition, replicate, timepoint, and perturbed prompt variant, the comparison against P00 was retained only when both the perturbed response and the matched P00 response were valid. Invalid outputs were recorded explicitly and were not zero-filled. For each retained pair, diagnosis divergence and action divergence were computed as Jensen-Shannon distances between the perturbed and matched baseline distributions [22,23]. The mean total Jensen-Shannon distance was defined as the average of the diagnosis and action Jensen-Shannon distance. Additional endpoints were top-action switch rate, invalid output rate, and agreement between the self-declared top action and the argmax of the returned action-probability distribution. Probability-vector validation was performed before distance calculation. Each diagnosis vector was required to contain 10 finite numeric non-negative values, and each action vector was required to contain eight finite numeric non-negative values. Because several models returned rounded probability values, otherwise valid vectors were not rejected solely because the raw pre-normalization sum differed from 1.0. Raw sums were logged, and vectors with positive total mass were normalized to sum to 1.0 before Jensen-Shannon distance calculation. Vectors with missing entries, non-numeric entries, negative values, non-finite values, or zero total mass were marked invalid. After normalization, accepted vectors were required to sum to 1.0 within a tolerance of 1e-6.

Statistical approach

This was a descriptive benchmark study rather than a patient-level inferential study. The design enumerated prespecified model, case, prompt, and condition cells within the chosen benchmark, and pair-valid filtering produced unequal denominators across cells. We therefore emphasized effect sizes, rates, and directional consistency rather than null-hypothesis significance testing. Interpretation focused on the magnitude and pattern of divergence, switching, and integrity failures across the fixed benchmark.

Data availability and reproducibility

The data and code supporting this study are available in Zenodo at https://doi.org/10.5281/zenodo.20079035. The archive contains analysis-ready tables, high-resolution figures, figure-regeneration scripts, validation materials, the local Ollama experiment set, full prompt templates, prompt variants, diagnosis/action ontologies, the JSON schema, sanitized Ollama model metadata, model-identification tables, quantization metadata, hardware metadata, and supplementary reproducibility text. All clinical cases are synthetic, and no patient data or protected health information are included.

## Results

Prompt sensitivity across model-condition cells

**Figure 2:**
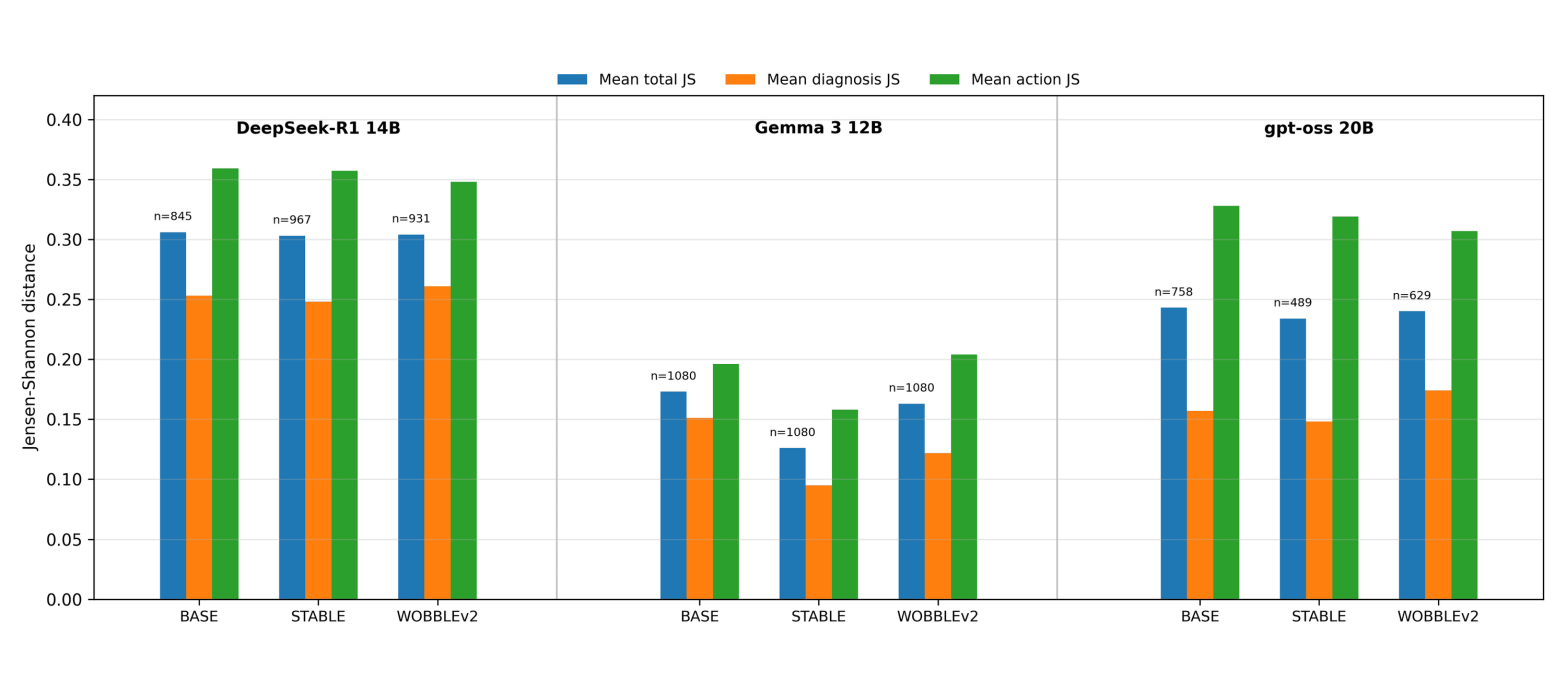
Overall prompt-induced divergence by model and condition. Grouped bars showing the mean total JS distance, the mean diagnosis JS distance, and the mean action JS distance for each model-condition cell. The mean total JS distance ranged from 0.126 to 0.306. In every cell, action divergence exceeded diagnosis divergence. STABLE substantially reduced divergence for Gemma 3 12B, but had only a minimal effect for DeepSeek-R1 14B and gpt-oss 20B. JS: Jensen-Shannon

Table 1 summarizes the overall divergence metrics by model and condition, and Figure 2 visualizes the same results as grouped bars. Across the nine model-condition cells, the mean total Jensen-Shannon distance did not approach zero. Values ranged from 0.126 to 0.306. The lowest mean divergence was observed for Gemma 3 12B under STABLE, and the highest was observed for DeepSeek-R1 14B under BASE. Gemma 3 12B showed the clearest benefit from stabilization: STABLE reduced the mean total Jensen-Shannon distance from 0.173 to 0.126, a relative reduction of 27.2%. By contrast, STABLE had only small effects for DeepSeek-R1 14B and gpt-oss 20B. WOBBLEv2 did not consistently exceed BASE in this local open model setting.

**Table 1:** Overall summary by model and condition. JS: Jensen-Shannon

Model	Condition	Mean total JS	Mean diagnosis JS	Mean action JS	T3 top-action switch	n
DeepSeek-R1 14B	BASE	0.306	0.253	0.359	0.471	845
DeepSeek-R1 14B	STABLE	0.303	0.248	0.357	0.533	967
DeepSeek-R1 14B	WOBBLEv2	0.304	0.261	0.348	0.531	931
Gemma 3 12B	BASE	0.173	0.151	0.196	0.389	1080
Gemma 3 12B	STABLE	0.126	0.095	0.158	0.253	1080
Gemma 3 12B	WOBBLEv2	0.163	0.122	0.204	0.433	1080
gpt-oss 20B	BASE	0.243	0.157	0.328	0.584	758
gpt-oss 20B	STABLE	0.234	0.148	0.319	0.529	489
gpt-oss 20B	WOBBLEv2	0.24	0.174	0.307	0.436	629

Diagnosis versus management sensitivity

Table 1 also shows that the mean action divergence exceeded the mean diagnosis divergence in every model-condition cell. The action-minus-diagnosis gap averaged 0.107 across the nine cells and ranged from 0.045 for Gemma 3 12B under BASE to 0.171 for gpt-oss 20B under both BASE and STABLE. At the terminal timepoint, top-1 action switching remained appreciable across all cells, ranging from 0.253 for Gemma 3 12B under STABLE to 0.584 for gpt-oss 20B under BASE. These findings indicate that local prompt sensitivity was not limited to changes in diagnostic labels and was particularly prominent for management choices.

Structured-output integrity

**Figure 3:**
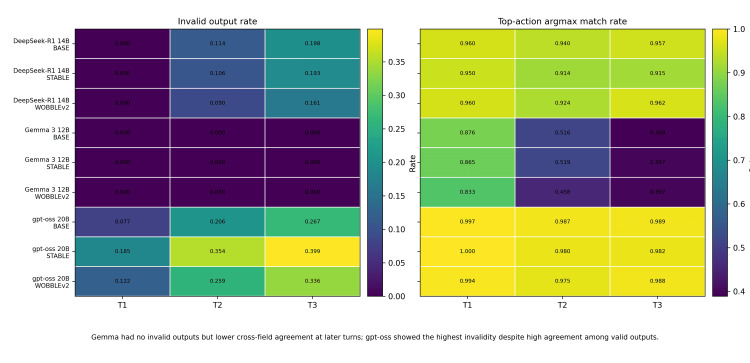
Integrity profiles by model, condition, and timepoint. Two-panel figure summarizing invalid output rate and top-action argmax match rate across T1-T3. Gemma 3 12B produced no invalid outputs in the primary analysis set, but agreement between the reported top action and the argmax of the returned action-probability vector fell markedly at later timepoints. By contrast, gpt-oss 20B maintained very high argmax agreement among valid outputs but exhibited the highest invalid-output rates, particularly under STABLE. DeepSeek-R1 14B showed intermediate integrity behavior on both axes.

Table 2 summarizes the invalid output rates and top-action argmax match rates, and Figure 3 visualizes these integrity profiles across timepoints. The three models displayed markedly different integrity behavior. Gemma 3 12B produced no invalid outputs in the primary analysis set, but its agreement between the self-declared top action and the argmax of action probabilities deteriorated over time, falling to 0.389-0.397 at the terminal timepoint. DeepSeek-R1 14B showed moderate invalidity that increased at later timepoints, reaching 0.198 under BASE and 0.193 under STABLE at the terminal timepoint, while maintaining high top-action argmax agreement. gpt-oss 20B showed the opposite trade-off: among valid outputs, top-action argmax agreement remained very high, but invalid rates were substantial and rose to 0.399 under STABLE at the terminal timepoint.

**Table 2:** Integrity summary by model, condition, and timepoint.

Model	Condition	Timepoint	Invalid rate	Top-action argmax match rate
DeepSeek-R1 14B	BASE	T1	0	0.96
DeepSeek-R1 14B	BASE	T2	0.114	0.94
DeepSeek-R1 14B	BASE	T3	0.198	0.957
DeepSeek-R1 14B	STABLE	T1	0	0.95
DeepSeek-R1 14B	STABLE	T2	0.106	0.914
DeepSeek-R1 14B	STABLE	T3	0.193	0.915
DeepSeek-R1 14B	WOBBLEv2	T1	0	0.96
DeepSeek-R1 14B	WOBBLEv2	T2	0.09	0.924
DeepSeek-R1 14B	WOBBLEv2	T3	0.161	0.962
Gemma 3 12B	BASE	T1	0	0.876
Gemma 3 12B	BASE	T2	0	0.516
Gemma 3 12B	BASE	T3	0	0.389
Gemma 3 12B	STABLE	T1	0	0.865
Gemma 3 12B	STABLE	T2	0	0.519
Gemma 3 12B	STABLE	T3	0	0.397
Gemma 3 12B	WOBBLEv2	T1	0	0.833
Gemma 3 12B	WOBBLEv2	T2	0	0.458
Gemma 3 12B	WOBBLEv2	T3	0	0.397
gpt-oss 20B	BASE	T1	0.077	0.997
gpt-oss 20B	BASE	T2	0.206	0.987
gpt-oss 20B	BASE	T3	0.267	0.989
gpt-oss 20B	STABLE	T1	0.185	1
gpt-oss 20B	STABLE	T2	0.354	0.98
gpt-oss 20B	STABLE	T3	0.399	0.982
gpt-oss 20B	WOBBLEv2	T1	0.122	0.994
gpt-oss 20B	WOBBLEv2	T2	0.259	0.975
gpt-oss 20B	WOBBLEv2	T3	0.336	0.988

Destabilizing prompt families

**Figure 4:**
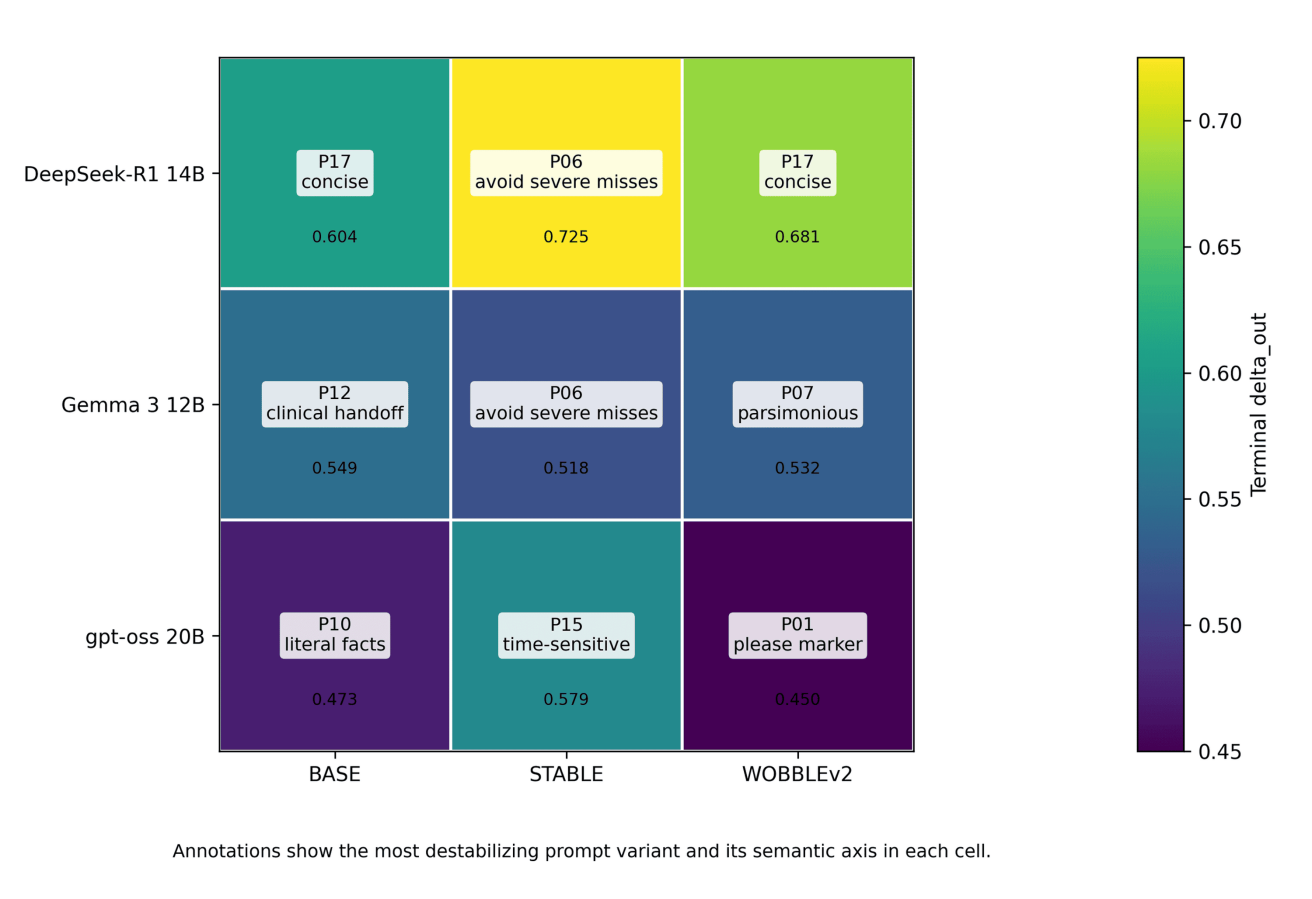
Most destabilizing prompt families across model-condition cells. Heatmap showing the prompt variant with the largest clean terminal delta_out in each model-condition cell. The most destabilizing variants were model-specific and spanned concise wording, risk-tolerance cues, clinical handoff framing, literal fact-use instructions, urgency cues, and politeness markers, underscoring that prompt sensitivity was driven by semantic framing rather than by punctuation alone.

Table 3 lists the most destabilizing prompt variant in each model-condition cell, and Figure 4 summarizes these prompt families as a heatmap. The variants with the largest terminal divergence were not uniform across models. For DeepSeek-R1 14B, concise wording and a severe-illness-avoidance cue were the strongest destabilizers. For Gemma 3 12B, clinical handoff framing and risk-tolerance framing were most destabilizing. For gpt-oss 20B, the largest effects came from literal fact-use instructions, time-sensitive framing, and a politeness marker. These findings indicate that prompt sensitivity in local models was semantically heterogeneous and model-specific.

**Table 3:** Most destabilizing prompt variant in each model-condition cell

Model	Condition	Prompt variant	Clean terminal delta_out	Family
DeepSeek-R1 14B	BASE	P17	0.604	Verbosity
DeepSeek-R1 14B	STABLE	P06	0.725	Risk tolerance
DeepSeek-R1 14B	WOBBLEv2	P17	0.681	Verbosity
Gemma 3 12B	BASE	P12	0.549	Genre
Gemma 3 12B	STABLE	P06	0.518	Risk tolerance
Gemma 3 12B	WOBBLEv2	P07	0.532	Risk tolerance
gpt-oss 20B	BASE	P10	0.473	Evidence policy
gpt-oss 20B	STABLE	P15	0.579	Urgency
gpt-oss 20B	WOBBLEv2	P01	0.450	Politeness

Timepoint progression

**Figure 5:**
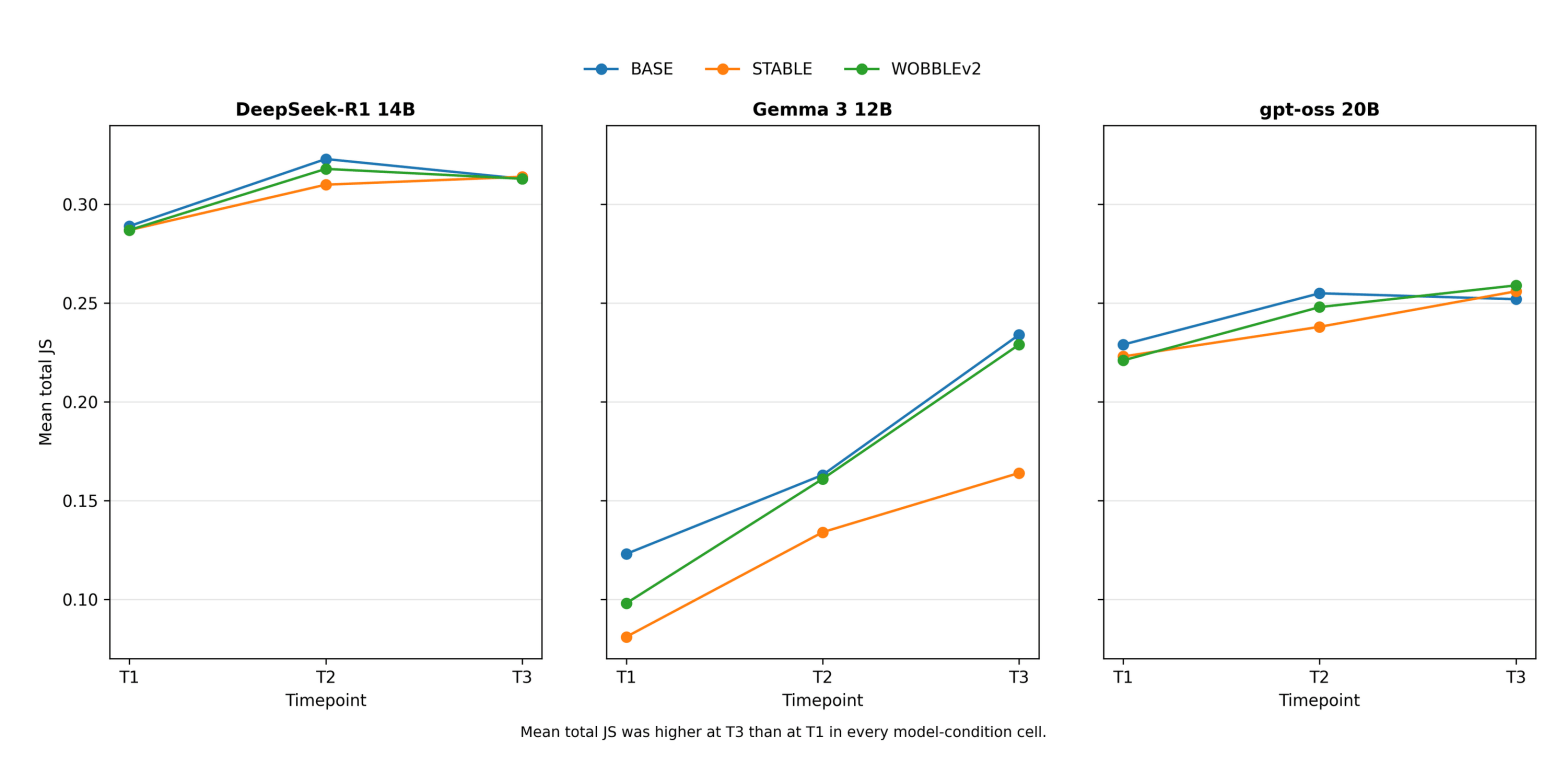
Time-amplified divergence across sequential clinical turns. Line plots of the mean total Jensen-Shannon distance across T1, T2, and T3 for each model under BASE, STABLE, and WOBBLEv2. In all nine model-condition cells, the mean total Jensen-Shannon distance was higher at T3 than at T1, indicating the accumulation of prompt-induced differences over time despite a fixed ontology and controlled timepoint disclosures.

Table 4 provides the timepoint-level overview of divergence and action switching, and Figure 5 displays the corresponding time-amplification curves. The mean total Jensen-Shannon distance was higher at the terminal timepoint than at the first timepoint in all nine cells. The increase was modest but consistent for DeepSeek-R1 14B and gpt-oss 20B. Gemma 3 12B showed larger amplification, with terminal-minus-initial increases of 0.083 under STABLE, 0.111 under BASE, and 0.132 under WOBBLEv2. In several cells, the trajectory was not perfectly monotonic at the intermediate turn, but the endpoint increase from the first to the terminal timepoint remained consistent.

**Table 4:** Timepoint overview of divergence and action switching. JS: Jensen-Shannon

Model	Condition	Total JS T1	Dx JS T1	Action JS T1	Switch T1	Total JS T2	Dx JS T2	Action JS T2	Switch T2	Total JS T3	Dx JS T3	Action JS T3	Switch T3
DeepSeek-R1 14B	BASE	0.289	0.218	0.359	0.503	0.323	0.26	0.385	0.813	0.313	0.3	0.327	0.471
DeepSeek-R1 14B	STABLE	0.287	0.232	0.341	0.425	0.31	0.25	0.37	0.697	0.314	0.265	0.363	0.533
DeepSeek-R1 14B	WOBBLEv2	0.287	0.234	0.339	0.408	0.318	0.264	0.372	0.571	0.313	0.293	0.333	0.531
Gemma 3 12B	BASE	0.123	0.107	0.139	0.042	0.163	0.135	0.192	0.208	0.234	0.211	0.256	0.389
Gemma 3 12B	STABLE	0.081	0.069	0.092	0.022	0.134	0.101	0.167	0.222	0.164	0.114	0.214	0.253
Gemma 3 12B	WOBBLEv2	0.098	0.069	0.126	0.039	0.161	0.106	0.217	0.311	0.229	0.19	0.268	0.433
gpt-oss 20B	BASE	0.229	0.144	0.314	0.314	0.255	0.162	0.347	0.502	0.252	0.175	0.329	0.584
gpt-oss 20B	STABLE	0.223	0.142	0.304	0.265	0.238	0.151	0.325	0.552	0.256	0.16	0.352	0.529
gpt-oss 20B	WOBBLEv2	0.221	0.153	0.288	0.152	0.248	0.188	0.309	0.343	0.259	0.186	0.331	0.436

## Discussion

Principal findings

This benchmark produced four main findings. First, measurable prompt sensitivity was present in every model-condition cell, including ordinary BASE and STABLE prompting. Second, management outputs were consistently more labile than diagnosis outputs. Third, prompt-induced divergence accumulated across sequential clinical turns. Fourth, structured-output robustness separated into at least two axes: distributional stability and output integrity. Together, these findings suggest that local deployment, schema constraints, and stabilizing prompt layers do not by themselves guarantee stable clinical reasoning outputs.

Interpretation of baseline sensitivity

Substantial baseline divergence is important because it challenges a common assumption in prompt robustness research. In constrained or user-interface-only settings, investigators may first observe an almost invariant baseline and then use stress conditions to elicit divergence. Our local results indicate that this stable operating regime may not transfer to deployments that combine open models, local serving stacks, and multi-turn interaction. Positive-control audit prompts remain valuable for pipeline validation, but the distinction between ordinary and stress-induced sensitivity may narrow when baseline sensitivity is already high. This interpretation is consistent with clinical evaluations showing degradation in decision-support performance when models must follow instructions, elicit information, or operate within more realistic workflows rather than answer self-contained examination-style items [8,13,19-21].

Why management divergence matters

The asymmetry between diagnosis and management is clinically important and aligns with evidence from richer clinical benchmarks. Clinical reasoning benchmarks suggest that diagnostic performance can remain relatively strong once sufficient information is available, whereas examination recommendation and treatment planning can remain weaker or less consistent [15,20]. One plausible explanation is that management distributions are higher-dimensional and more threshold-dependent than diagnosis distributions. A model can converge on what is happening while remaining unstable on what should happen next, especially when prompts shift implied risk tolerance, urgency, or evidentiary style. For bedside decision support, this distinction may matter more than the final diagnostic label alone.

Dialogue-level robustness

The temporal pattern supports interpreting prompt sensitivity as a dialogue-level robustness phenomenon rather than a single-turn formatting artifact. Clinical reasoning unfolds through sequential updates as new information arrives, and prior studies have shown that LLM behavior can change with the order, amount, and interactive history of information presented [8,13,14]. Our data provide a complementary view: even when facts disclosed at each turn are held constant, small initial wording differences can persist and amplify across turns. We interpret this pattern as time-amplified prompt sensitivity rather than as evidence of formal mathematical chaos. The result underscores that robustness should be audited over trajectories, not only at isolated snapshots.

Distributional stability and output integrity as separate axes

The integrity findings suggest that robustness in local clinical LLM workflows is at least two-dimensional. One axis captures distributional stability, meaning how much probability mass over diagnoses or actions shifts under prompt variation. A second axis captures structured-output integrity, meaning whether the model preserves parseability and internal agreement among output fields. Health-artificial-intelligence evaluation frameworks increasingly emphasize multidimensional assessment that includes risk, usability, reproducibility, and implementation constraints, not accuracy alone [9-12]. Our results provide an operational example of this point. A model can be syntactically reliable yet internally inconsistent within its own structured fields, as observed with Gemma 3 12B. Conversely, a model can be internally coherent when valid yet fail too often at the formatting layer, as observed with gpt-oss 20B. Schema-constrained generation therefore addresses only part of the problem.

Semantic prompt families and model specificity

The model-specific destabilizing prompt families fit broader prompt robustness literature, which emphasizes that semantically meaningful perturbations cannot be captured by character-level distance alone [6,7]. In clinical contexts, these semantic axes have realistic analogues. Handoff style reflects how clinicians summarize cases across settings; urgency cues mirror emergency department or intensive care communication; evidence-policy instructions resemble requests for literal versus inferential reasoning; and risk-tolerance cues approximate differences in defensive versus parsimonious practice styles. Our model-specific findings suggest that a stability layer is not a universal regularizer. Rather, it acts as a model-specific control input whose effect depends on each model's post-training, instruction-following priors, and reasoning style.

Practical implications

Several operational lessons follow from these results. Pair-valid analysis should be preferred over zero-filling invalid responses because parse failures can otherwise masquerade as extreme semantic divergence. Top-label fields should not be trusted without recomputation from returned probability vectors; the Gemma 3 12B results show that JSON validity alone is insufficient. Stability prompts should be validated separately for each model family rather than assumed to transfer. In our data, STABLE functioned as a meaningful regularizer for Gemma 3 12B but offered little benefit for DeepSeek-R1 14B and gpt-oss 20B. Trajectory-level testing is also necessary because single-turn prompt checks would have underestimated the later amplification observed in every model-condition cell.

These recommendations align with broader calls for reproducible, clinician-aware evaluation of generative artificial intelligence in medicine [9-12]. They also suggest a practical triage framework for local use. Models with high invalidity may require retry, fallback, or parser-repair pipelines before downstream scoring. Models with low invalidity but poor cross-field agreement may require semantic consistency audits before outputs are shown to users. Models whose management distributions are especially volatile may warrant restricted use cases, such as documentation assistance, rather than unreviewed escalation or treatment planning.

Limitations and future work

This study has several limitations. First, all cases were synthetic and evaluated within a fixed ontology, so external clinical validity is limited. Second, the primary analysis used six cases and three timepoints; longer trajectories or broader case libraries may reveal additional sensitivity patterns. Third, the study was descriptive and did not include clinician adjudication, calibration analysis, or an oracle-regret framework. Fourth, the action ontology was intentionally coarse and may under-represent model differences for procedural edge cases. Fifth, pair-valid analysis summarizes divergence only among retained valid pairs; in cells with high invalidity, semantic divergence may therefore be underestimated unless interpreted together with integrity endpoints. Sixth, the observed patterns reflect the joint model-plus-serving-stack configuration and may change with different quantization, runtime, parser, or schema-enforcement settings. Seventh, broader stress-sweep analyses, including gain-based forcing and cross-language comparisons, were outside the scope of the current paper. Finally, because the benchmark used schema-constrained outputs, absolute robustness levels may differ from unconstrained free-text settings.

Future work should extend this framework in at least four directions. Clinician labeling is needed to separate medically acceptable branching from hazardous divergence. Uncertainty and calibration analyses should be incorporated so that prompt sensitivity can be related to the model's ability to communicate uncertainty appropriately [16,17]. Belief-state or oracle-regret simulators could connect output divergence to downstream utility more directly. Longer-horizon, multilingual, and partially observed dialogue environments may better reflect the contexts in which local models would be used.

## Conclusions

In this multi-model local benchmark, prompt sensitivity was measurable across all evaluated model-condition cells, including ordinary BASE and STABLE prompting. Management distributions were consistently more labile than diagnostic distributions, timepoint progression amplified divergence, and structured-output integrity failures varied sharply across model families. These results suggest that local deployment does not guarantee stable prompt-response behavior and that robustness audits for clinical LLM workflows should report both distributional divergence and output integrity.

For local clinical use, the central question is not only whether a model can generate a plausible answer but whether it does so consistently across ordinary variations in wording, framing, and sequential dialogue context. The accompanying dataset and code archive are intended to support reproducibility and external testing of this audit approach.

## References

[1] Singhal K, Azizi S, Tu T (2023). Large language models encode clinical knowledge. Nature.

[2] Singhal K, Tu T, Gottweis J (2025). Toward expert-level medical question answering with large language models. Nat Med.

[3] Kung TH, Cheatham M, Medenilla A (2023). Performance of ChatGPT on USMLE: potential for AI-assisted medical education using large language models. PLOS Digit Health.

[4] Thirunavukarasu AJ, Ting DS, Elangovan K, Gutierrez L, Tan TF, Ting DS (2023). Large language models in medicine. Nat Med.

[5] Meng X, Yan X, Zhang K (2024). The application of large language models in medicine: a scoping review. iScience.

[6] Liu P, Yuan W, Fu J, Jiang Z, Hayashi H, Neubig G (2023). Pre-train, prompt, and predict: a systematic survey of prompting methods in natural language processing. ACM Comput Surv.

[7] Sivarajkumar S, Kelley M, Samolyk-Mazzanti A, Visweswaran S, Wang Y (2024). An empirical evaluation of prompting strategies for large language models in zero-shot clinical natural language processing: algorithm development and validation study. JMIR Med Inform.

[8] Hager P, Jungmann F, Holland R (2024). Evaluation and mitigation of the limitations of large language models in clinical decision-making. Nat Med.

[9] Livingston L, Featherstone-Uwague A, Barry A, Barretto K, Morey T, Herrmannova D, Avula V (2025). Reproducible generative artificial intelligence evaluation for health care: a clinician-in-the-loop approach. JAMIA Open.

[10] Denniston AK, Liu X (2024). Responsible and evidence-based AI: 5 years on. Lancet Digit Health.

[11] Marconi L, Cabitza F (2025). Show and tell: a critical review on robustness and uncertainty for a more responsible medical AI. Int J Med Inform.

[12] Jacob C, Brasier N, Laurenzi E, Heuss S, Mougiakakou SG, Cöltekin A, Peter MK (2025). AI for IMPACTS framework for evaluating the long-term real-world impacts of AI-powered clinician tools: systematic review and narrative synthesis. J Med Internet Res.

[13] McDuff D, Schaekermann M, Tu T (2025). Towards accurate differential diagnosis with large language models. Nature.

[14] Tu T, Schaekermann M, Palepu A (2025). Towards conversational diagnostic artificial intelligence. Nature.

[15] Qiu P, Wu C, Liu S (2025). Quantifying the reasoning abilities of LLMs on clinical cases. Nat Commun.

[16] Savage T, Wang J, Gallo R (2025). Large language model uncertainty proxies: discrimination and calibration for medical diagnosis and treatment. J Am Med Inform Assoc.

[17] Zhou S, Wang J, Xu Z (2025). Uncertainty-aware large language models for explainable disease diagnosis. NPJ Digit Med.

[18] Wu C, Qiu P, Liu J (2025). Towards evaluating and building versatile large language models for medicine. NPJ Digit Med.

[19] Sandmann S, Hegselmann S, Fujarski M, Bickmann L, Wild B, Eils R, Varghese J (2025). Benchmark evaluation of DeepSeek large language models in clinical decision-making. Nat Med.

[20] Vrdoljak J, Boban Z, Males I (2025). Evaluating large language and large reasoning models as decision support tools in emergency internal medicine. Comput Biol Med.

[21] Sim SZ, Chen T (2025). Critique of impure reason: unveiling the reasoning behaviour of medical large language models [Preprint]. arXiv.

[22] Lin J (1991). Divergence measures based on the Shannon entropy. IEEE Transactions on Information Theory.

[23] Endres DM, Schindelin JE (2003). A new metric for probability distributions. IEEE Transactions on Information Theory.

